# Supplementary material for: Comparative genomics provides new insights into the diversity, physiology, and sexuality of the only industrially exploited tremellomycete: Phaffia rhodozyma
Source: BMC Genomics. 2016 Nov 9;17:901. doi: 10.1186/s12864-016-3244-7 (PMC5103461; doi:10.1186/s12864-016-3244-7)
Supplement: Additional file 6: — List of orphan genes with links to PFAM (related to Additional file 1: Table S1). (ZIP 1428 kb) [file 12864_2016_3244_MOESM6_ESM.zip › PFAM_UNIQUE/G02428_P.html]

#                                                                            --- full sequence --- -------------- this domain -------------   hmm coord   ali coord   env coord
  
# target name        accession   tlen query name           accession   qlen   E-value  score  bias   #  of  c-Evalue  i-Evalue  score  bias  from    to  from    to  from    to  acc description of target
  
#------------------- ---------- ----- -------------------- ---------- ----- --------- ------ ----- --- --- --------- --------- ------ ----- ----- ----- ----- ----- ----- ----- ---- ---------------------
  
G02428\_P             -            226 CDC27                PF09507.5    430         4    5.2   7.9   1   1     0.032         5    4.9   7.9   193   272    14    90     6   131 0.58 - DNA polymerase subunit Cdc27  
G02428\_P             -            226 FLO\_LFY              PF01698.11   387      0.93    6.9   4.5   1   1    0.0031       1.5    6.2   4.5   144   222    12    92     2   102 0.45 - Floricaula / Leafy protein  
G02428\_P             -            226 Gasdermin            PF04598.7    456     0.017   12.5   0.5   1   1   7.2e-06     0.022   12.2   0.5   215   402    37   209    35   219 0.83 - Gasdermin family  
G02428\_P             -            226 Lin-8                PF03353.10   313      0.24    9.3   6.3   1   1   0.00086      0.32    8.9   6.3   127   283    23   169    17   176 0.72 - Ras-mediated vulval-induction antagonist  
G02428\_P             -            226 MIP-T3               PF10243.4    539       4.3    4.3   6.2   1   1     0.038       4.9    4.1   6.2   215   288    26   103     6   167 0.59 - Microtubule-binding protein MIP-T3  
G02428\_P             -            226 ORC6                 PF05460.8    353      0.17    9.6   2.7   1   1   0.00072       0.2    9.4   2.7   105   162    14   116     2   177 0.62 - Origin recognition complex subunit 6 (ORC6)  
G02428\_P             -            226 Prothymosin          PF03247.9    106       3.5    6.5   6.3   1   1     0.043       5.7    5.8   6.3    17    62    51    95    27   106 0.70 - Prothymosin/parathymosin family  
G02428\_P             -            226 PTRF\_SDPR            PF15237.1    246      0.28    9.2   7.1   1   1     0.001      0.67    8.0   7.1   100   161    32   108    25   155 0.66 - PTRF/SDPR family  
G02428\_P             -            226 SART-1               PF03343.8    613      0.18    8.7   2.7   1   1    0.0011      0.18    8.7   2.7   252   323    26    89     3   202 0.54 - SART-1 family  
G02428\_P             -            226 THRAP3\_BCLAF1        PF15440.1    646       6.8    3.9   7.2   1   1     0.012         8    3.6   7.2   186   638    23    89     3   133 0.61 - THRAP3/BCLAF1 family  
G02428\_P             -            226 U79\_P34              PF03064.11   251       6.9    4.8   7.6   1   1     0.015       9.1    4.4   7.6   160   229    48   113    14   128 0.62 - HSV U79 / HCMV P34  
G02428\_P             -            226 UPF0564              PF10595.4    356      0.48    7.9   3.3   1   1    0.0024      0.58    7.7   3.3   173   253    21    97     5   165 0.69 - Uncharacterised protein family UPF0564
